# Something Right about Catgut*A Lecture at the N. Y. Post-Graduate Medical School, Oct. 21, 1897.

**Published:** 1898-01

**Authors:** Robert T. Morris

**Affiliations:** New York City


### Selections and Abstracts.

### SOMETHING RIGHT ABOUT CATGUT.*

By ROBERT T. MORRIS, M. D., New York City.

Several major points and several minor points about catgut will be remembered more easily if we group them separately.

Major Point No. 1.—When buying catgut order the sizes by American standard wire gauge. Different dealers have an arbitrary numbering of sizes and that causes a great deal of confusion. An American standard wire gauge can be found in the hardware shops and in mechanics' work-rooms—and if you order sizes by that definite measurement, the dealer who receives the order can step around the corner and invest a half dollar in a proper little wire gauge which tells him how to number catgut accurately.

Major Point No. 2.—The form of catgut is important because of the ease or difficulty in handling it.

I prefer the sort sold by dealers in watchmakers' supplies. It is used for their bow drills, and goes by the name of "bow lines." Each bow line is one metre in length. As many strands as we choose can be removed from the bottle at the time of an operation, without contaminating the remainder.

Major Point No. 3.—Catgut is satisfactorily prepared by the formal process. The word "formal" is the proper contraction for the word "formalin." The word "formalin" is the trade term for a commercial preparation of a forty per cent. solution of formaldehyde gas in water. A two per cent. solution of this commercial formalin is what we mean when speaking of a two per cent. solution of formal.

Major Point No. 4.—Catgut, raw, oily and dirty, is placed in a jar of water containing two per cent. of formal. The bulk of the water should be three or four times the bulk of the catgut. The catgut remains in this solution for forty-eight hours, and by the end of that time it has become hardened and sterile. It can then be boiled in water without suffering damage if the formal is removed.

Major Point No. 5.—Formal is irritating to the tissues and it destroys catgut unless it is removed. Wash out the formal by putting the catgut in a bowl and letting water from the faucet run through it for twelve hours. Better attach a rubber tube to the faucet and drop one end of the tube in the bottom of the bowl of catgut so that water escapes upward

* A Lecture at the N. Y. Post-Graduate Medical School, Oct. 21, 1897.

through the mass of catgut—carrying formal with the outflowing current. The catgut which was hardened and sterilized is now contaminated by the tap water and it needs to be boiled in water for final sterilization.

Major Point No. 6.—Boil the catgut in water for fifteen minutes.

Major Point No. 7.—Put the boiled catgut in absolute alcohol and use it directly from the alcohol at time of operation.

Minor Point A.—Catgut prepared by the formal process resists absorption in the tissues in proportion to diameter. We may expect that No. 16 American standard wire gauge will resist complete absorption for three weeks, while No. 26 will be absorbed in about a week.

Minor Point B.—If you wish to prepare a lot of catgut with greater resistance to absorption, add one-quarter of one per cent. of bichromate of potassium to the formal solution.

Minor Point C.—Catgut does not dry so quickly on removal from the alcohol if five per cent. of glycerine is added to the alcohol.

Minor Point D.—Catgut placed in alcohol immediately after being boiled carries a good deal of water in its tissue, consequently it is better to abstract the water by placing the catgut in one jar of alcohol for a day and then transferring the catgut to another lot of alcohol for permanent storage.

Minor Point E.—Catgut twists into knots while it is being boiled in water. To prevent this, bunches about the size of hens' eggs are firmly rolled in a gauze bandage and the bandage is tightly pinned before the lot is thrown into boiling water.

Minor Point F.—Boiled formal catgut shrinks to about two-thirds of its former length, and is too elastic to be used with comfort. To obviate this, a strand before being used is pulled out to its original length with the hands, when it suddenly loses its elasticity and remains at its original length.

Minor Point G.—If catgut has been taken out of the alcohol bottle and not used at an operation, boil it again before replacing it in the bottle.

For fourteen years I have depended upon catgut for use in all of my work excepting in certain bone surgery which required the employment of silver wire.

During these fourteen years I have occasionally taken up other materials for sutures and ligatures, but have quickly discarded such materials for catgut again. *Kangaroo tendon* seems to be as good as the best catgut, but it is more expensive and the sources of supply can not be depended upon. I used up a lot kindly given me by Dr. Marcy, and found it excellent, but no better than my own good catgut.

*Silk* often works out of a wound or scar slowly and causes troublesome sinuses. I have at present in the hospital a patient whose buried silk sutures have been gradually coming out of an abdominal scar for several years. The excursions of silk sutures are well known. The idea that silk knots hold better than catgut knots is advocated principally by men who tie granny knots. The first thing that a surgeon should learn is the tying of a square knot—but I have seen granny knots tied by at least one distinguished surgeon.

*Horse hair*, *linen*, *Chinese grass* and various other suture materials are advocated from time to time by men who do not prepare catgut well.

*Silkworm gut* is an instrument of the devil when used for buried permanent sutures. About three years ago I read an article on the use of silkworm gut by a surgeon whom I knew to be a careful observer, and I was tempted to give up my good-enough catgut for something better. In the course of six weeks I employed buried silkworm gut sutures in some twenty abdominal operations for patients who had put confidence in me and had entrusted their lives to my care. At the end of the six weeks I found that some of these buried silk knots were coming out in strange places and some of them are coming out yet—three years elapsed. One patient died as the result of the burrowing of a silkworm gut knot in the pelvis several months after operation. Nearly all of the patients suffered in one way or another. The surgeon who wrote the paper advocating the use of the buried silkworm gut knot has written another paper saying that he is sorry. Meanwhile, other surgeons are bringing discredit upon surgery by taking up this innocent-looking suture material which is so smooth and nice but which refuses to become encapsulated in the tissues, and which comes out of the bladder or iliac vein just after the surgeon has written his paper. A recent advocate of the buried silkworm gut suture intimates that some of us did not use aseptic precaution, and that we did not choose the right sort of silkworm gut. To this I will reply that most of my wounds healed completely by primary union and the knots came out months or years afterward. Further, as a fisherman, I am fully conversant with the subject of silkworm gut and have not been deceived in the quality of the material used. To those who say they have had no trouble with buried silkworm gut knots I wish to say that they have not taken the trouble to find out. The trouble is all on the part of the patients. The patient goes to one surgeon for operation, and to another one to complain. So it requires two surgeons to write the history of buried silkworm gut.—*New England Medical Monthly*.

